# Epigenetic clock and methylation study of oocytes from a bovine model of reproductive aging

**DOI:** 10.1111/acel.13349

**Published:** 2021-04-02

**Authors:** Paweł Kordowitzki, Amin Haghani, Joseph A. Zoller, Caesar Z. Li, Ken Raj, Matthew L. Spangler, Steve Horvath

**Affiliations:** ^1^ Institute of Animal Reproduction and Food Research of Polish Academy of Sciences Olsztyn Poland; ^2^ Institute for Veterinary Medicine Nicolaus Copernicus University Torun Poland; ^3^ Department of Human Genetics David Geffen School of Medicine University of California Los Angeles CA USA; ^4^ Department of Biostatistics Fielding School of Public Health University of California Los Angeles CA USA; ^5^ Radiation Effects Department Centre for Radiation, Chemical and Environmental Hazards Public Health England Didcot UK; ^6^ Department of Animal Science University of Nebraska Lincoln NE USA

**Keywords:** DNA methylation, epigenetic clock, epigenome‐wide association study, oocytes, reproductive aging

## Abstract

Cattle are an attractive animal model of fertility in women due to their high degree of similarity relative to follicle selection, embryo cleavage, blastocyst formation, and gestation length. To facilitate future studies of the epigenetic underpinnings of aging effects in the female reproductive axis, several DNA methylation‐based biomarkers of aging (epigenetic clocks) for bovine oocytes are presented. One such clock was germane to only oocytes, while a dual‐tissue clock was highly predictive of age in both oocytes and blood. Dual species clocks that apply to both humans and cattle were also developed and evaluated. These epigenetic clocks can be used to accurately estimate the biological age of oocytes. Both epigenetic clock studies and epigenome‐wide association studies revealed that blood and oocytes differ substantially with respect to aging and the underlying epigenetic signatures that potentially influence the aging process. The rate of epigenetic aging was found to be slower in oocytes compared to blood; however, oocytes appeared to begin at an older epigenetic age. The epigenetic clocks for oocytes are expected to address questions in the field of reproductive aging, including the central question: how to slow aging of oocytes.

## Graphical abstract

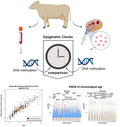

## 1 INTRODUCTION

The mammalian female reproductive axis is the first to fail in aging; however, the molecular mechanisms underpinning this failure are largely unknown, particularly in oocytes (Chamani & Keefe, 2019; Garg & Sinclair, 2015; Homer, 2020). Fertility in women begins to decline significantly by their mid‐30 s and pregnancies in women of advanced age lead to higher rates of miscarriage and/or aneuploid offspring (Baired et al., 2005). Despite these risks, women and couples often postpone pregnancy to a more convenient time which has led to a decline in birthrate among most industrialized societies (Homer, 2020; Navot et al., 1991). This decline in fertility can be explained by the age‐related decline in oocyte quality which manifests itself by chromosomal abnormalities, spindle defects, mitochondrial dysfunction, and epigenetic modifications (Baired et al., 2005; Kikuchi et al., 2000; Liu et al., 2002; López‐Otín et al., 2013; Pellestor et al., 2003; Xu et al., 1997). Due to ethical restrictions for research on human oocytes, bovine oocytes are a common and attractive model of oocyte developmental competence and reproduction. Interest in bovine pre‐implantation embryology and bovine/ruminant in vitro models in the field of human reproduction has increased (Adams et al., 2012; Hyttel et al., 2019).

Oocytes of donors with increased maternal age also show aberrant global DNA methylation levels (Marshall & Rivera, 2018). Previous work has shown that highly accurate estimators of chronological age (epigenetic clocks) that apply to most tissues and cell types can be developed using DNA methylation levels at individual loci (Horvath, 2013; Petkovich et al., 2017). The current study addresses several key questions surrounding the development of an epigenetic clock of oocytes. Since oocytes can be readily collected in cattle, DNA from both cattle and humans was used to address the following aims: (1) to test whether an epigenetic clock that applies to blood also applies to oocytes, (2) to build an epigenetic clock for bovine oocytes, (3) to build epigenetic clocks that apply to both cattle and humans to facilitate translation of research findings, (4) to evaluate whether epigenetic age acceleration detected in blood is also manifested in oocytes, and (5) to ascertain the association between age and methylation levels of individual CpGs in oocytes.

## 2 RESULTS

**FIGURE 1:**
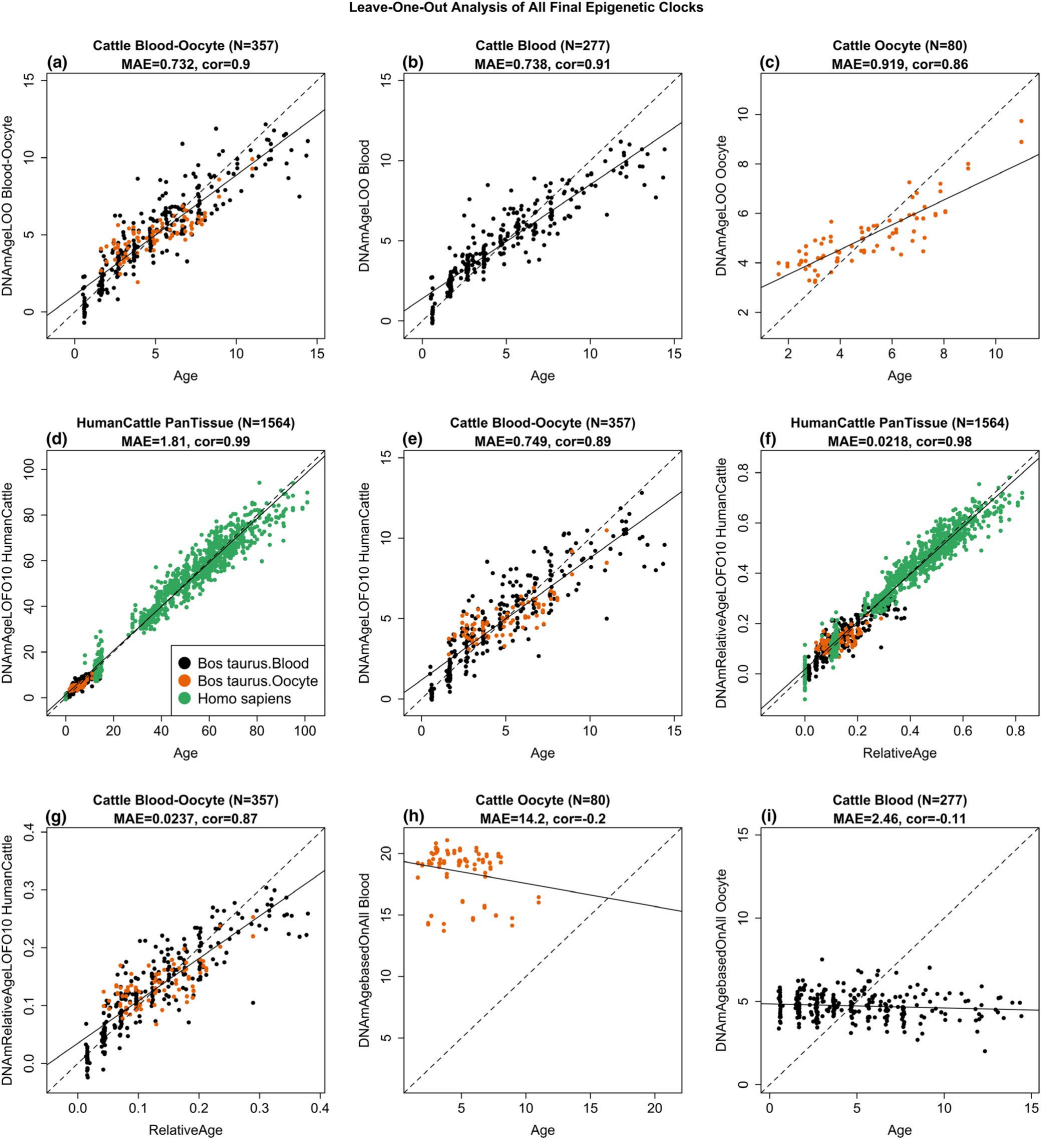
Cross‐validation study of epigenetic clocks for cattle and humans. (a) Blood‐oocyte clock for cattle applied to blood and oocytes, (b) Cattle clock for blood, (c) Cattle clock for oocytes. Chronological age (*x*‐axis, in units of years) versus the leave‐one‐sample‐out (LOO) estimate of DNA methylation age (y‐axis, in units of years). Dots are colored by tissue type (orange = oocytes, black = blood). (d, e) Ten‐fold cross‐validation analysis of the human‐cattle clock for (chronological) age applied (d) to samples from both species and to (e) to cattle only. (f, g) Human‐cattle clock estimate of relative age defined as the ratio of chronological age to the maximum life span of the respective species. Ten‐fold cross‐validation estimates of age (*y*‐axis, in years) in (d, f) Human (green) and cattle (orange) samples and (e, g) cattle samples only (colored by tissue type). Each panel reports the sample size, correlation coefficient, median absolute error (MAE). (h) Cattle blood clock applied to oocytes from cattle. (i) Cattle oocyte clock applied to blood samples from cattle

DNA methylation data were generated from DNA samples (*n* = 357) from oocyte donors (*n* = 80) and blood donors (*n* = 277) of cattle (*Bos taurus*). Bisulfite conversion was successful (on average 95%) for both blood and oocyte samples according to the quality control probes on the mammalian methylation array. Animals were between 0.5 years and 13.3 years of age at the time of tissue sampling. Unsupervised hierarchical clustering revealed that oocytes and blood methylation profiles form distinct clusters (Figure S3). Using penalized regression, several highly accurate epigenetic clocks were developed for cattle blood, cattle oocytes, and both tissues combined (Figure 1a‐c). These epigenetic clocks differ with regards to their operational parameters; tissue, species and measure of age.

To develop dual species clocks, DNA methylation profiles from human samples (*n* = 852) were added to the training set. Methylation data for both bovine and human tissues were generated from the HorvathMammalMethylChip40 array that consists of approximately 36,000 CpGs embedded in DNA sequences that are highly conserved within the mammalian class. As would be expected, a high percentage (45%) of conserved genes and regions between cattle and humans was found (Figure S1). The mammalian array coverage of the cattle genome (*Bos taurus*) ARS‐UCD1.2 based on genome alignments with the human Hg38 genome is reported herein (Figure S1). In total, 31,252 out of 37,540 probes mapped to the cattle genome and approximately 71% of these mapped to the same genes in cattle and humans (Figure S1). This high degree of conservation suggests that building dual species clocks that apply to both humans and cattle is indeed possible. The two human‐cattle clocks that were derived used the same set of CpGs and the same prediction equation to estimate age in both species. The dual species clocks presented herein can be distinguished by the measure of age; whereby one operates with chronological age in units of years, while the other employs relative age, which is the ratio of chronological age to the maximum lifespan of the species, and is expressed as values between 0 and 1. The maximum ages of cattle and humans are 38 years and 122.5 years, respectively. The high accuracy of the human‐cattle clock for relative age (Figure 1f,g) shows that this clock facilitates a biologically meaningful comparison between species with different life spans (cattle and human). The human‐cattle clock generated a correlation of *r* = 0.99 between chronological age and epigenetic age when both species were analyzed together (Figure 1d), but the correlation was slightly lower when the analysis was restricted to bovine oocytes and blood samples (*r* = 0.89, Figure 1e). The results of the human‐cattle clock for relative age show a comparably high correlation regardless of whether the analysis was performed with samples from both species (*r* = 0.98, Figure 1f) or only with bovine samples (*r* = 0.87, Figure 1g). By construction, the multi‐tissue clock for blood and oocytes applies to both sources of DNA (Figure 1a). Interestingly, the cattle clock for blood does not apply to oocytes (Figure 1h) and, conversely, the cattle clock for oocytes does not apply to cattle blood (Figure 1i).

**FIGURE 2:**
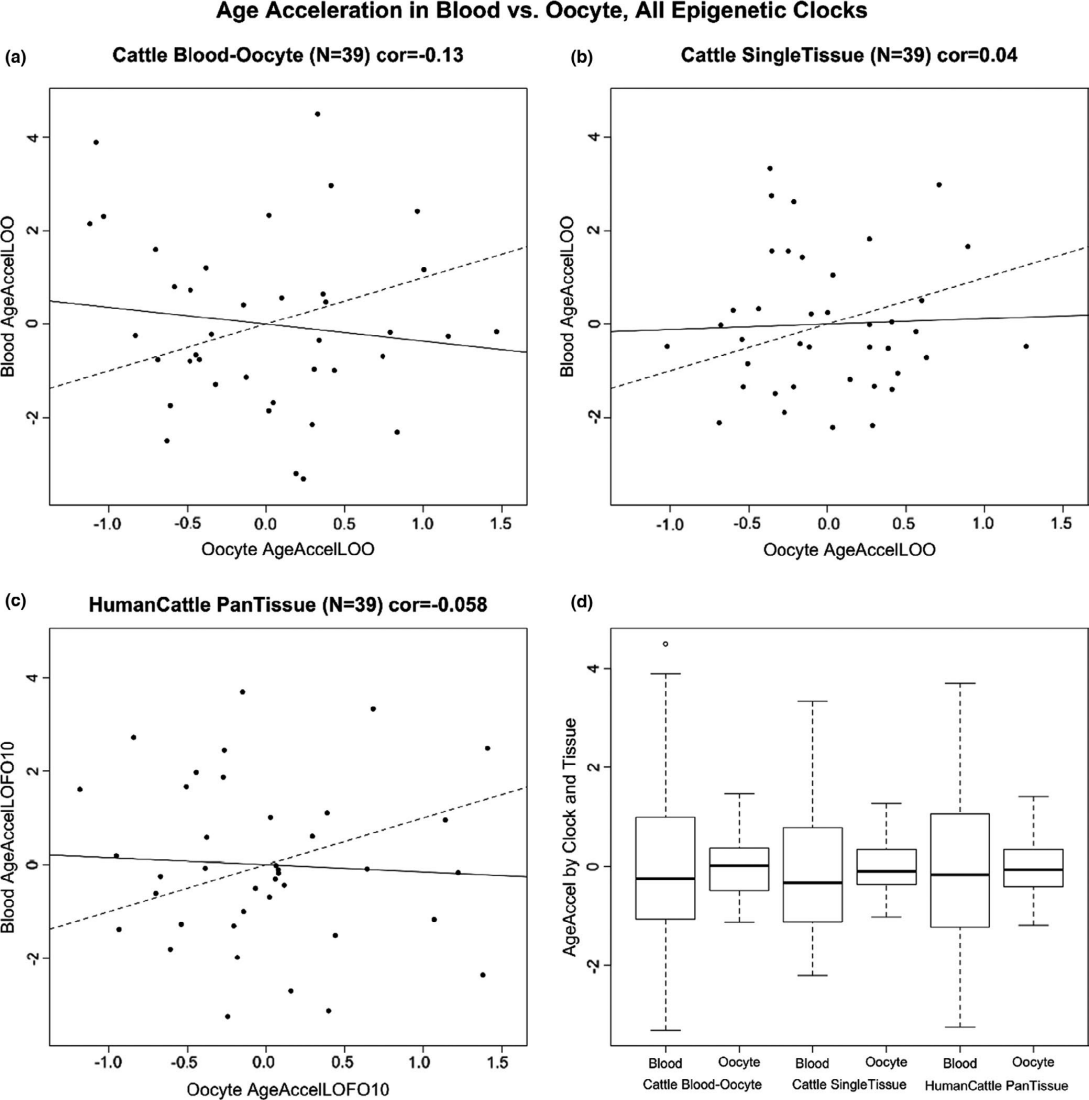
Epigenetic age acceleration in cattle blood is not correlated with that in oocytes. (a‐b) Cross‐validation estimates of epigenetic age acceleration in blood versus epigenetic age acceleration in oocytes. Dashed line indicates the diagonal line (*y* = *x*). The solid line corresponds to the regression line. (a) Results for the multi‐tissue cattle clock. (b) Single‐tissue clocks for oocytes (*x*‐axis) and blood (*y*‐axis). (c) Human‐cattle clock of chronological age. (d) Boxplots of epigenetic age acceleration in blood or oocytes for different cattle clocks

As a subset of data were generated from blood and oocytes from the same animal, epigenetic ages between these two isogenic tissues were directly compared. To avoid confounding by estrous phase, the samples were collected during a single estrous phase: the luteal phase. To avoid the confounding effect of chronological age, estimated DNAm age acceleration was used as the feature of comparison for both tissues. None of the epigenetic clocks resulted in a significant positive correlation between epigenetic age acceleration in blood and that of oocytes from the same cattle (Figure 2).

The epigenetic clocks for cattle performed poorly when used to predict the age of human blood and skin samples (Figure S4). The best performing of the three pure cattle clocks was the multi‐tissue clock whose age estimates were only moderately correlated with age in human blood (*r* = 0.32) and skin (*r* = 0.5) samples (Figure S4A,D). As expected, the human‐cattle clocks led to substantially higher age correlations in human samples (cross‐validation estimates of *r* = 0.97 in both blood and skin, Figure S5).

**FIGURE 3:**
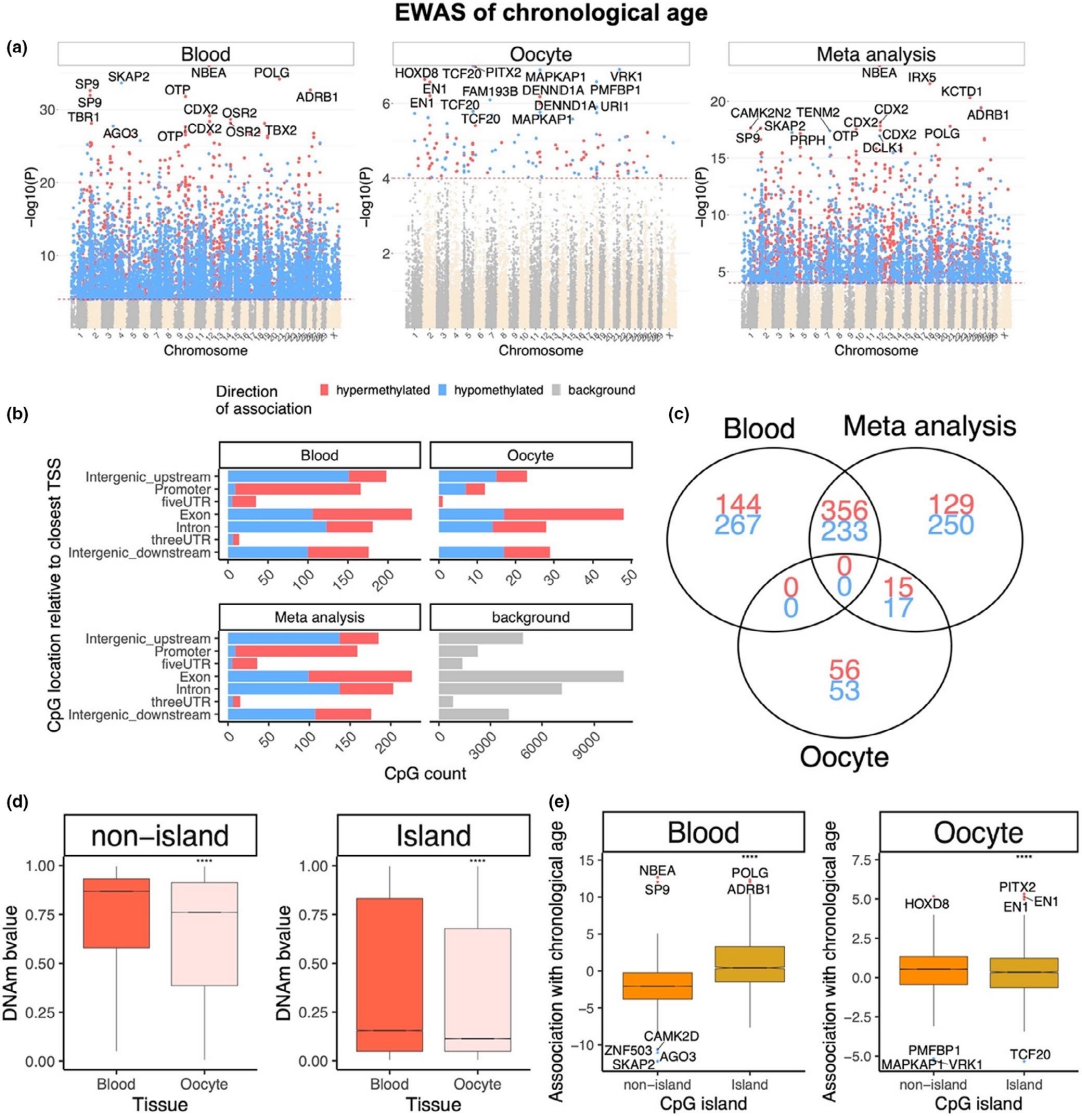
Blood and oocyte have distinct age‐dependent DNA methylation changes. (a) Manhattan plots of the EWAS of chronological age. The coordinates are estimated based on the alignment of Mammalian array probes to the Bos_taurus.ARS‐UCD1.2 genome assembly. The red dotted line corresponds to a significance threshold of *p* < 10^−4^. Individual CpGs are colored in red or blue if they gain or lose methylation with age. The 15 most significant CpGs are labeled by neighboring genes. (b) Location of top CpGs in each tissue is relative to the closest transcriptional start site. Top CpGs were selected (*p* < 10^−4^) and further filtering based on *z* score of association with chronological age for a maximum 500 in each direction (positive and negative). The number of selected CpGs: blood, 1000; oocyte, 141; meta‐analysis, 1000. The gray color in the last panel represents the location of 31,252 mammalian BeadChip array probes mapped to Bos_taurus.ARS‐UCD1.2 genome. (c) Venn diagram representing the overlap of aging‐associated CpGs based on meta‐analysis or individual tissues. (d) Comparison of DNAm by CpG island status in blood and. Oocytes have generally lower DNAm levels in island and non‐island CpGs than blood. (e) Boxplot of age correlation test Z statistics versus CpG island status in blood and oocytes. The mean difference was examined by *t* test. ****p < 1e‐4

**FIGURE 4:**
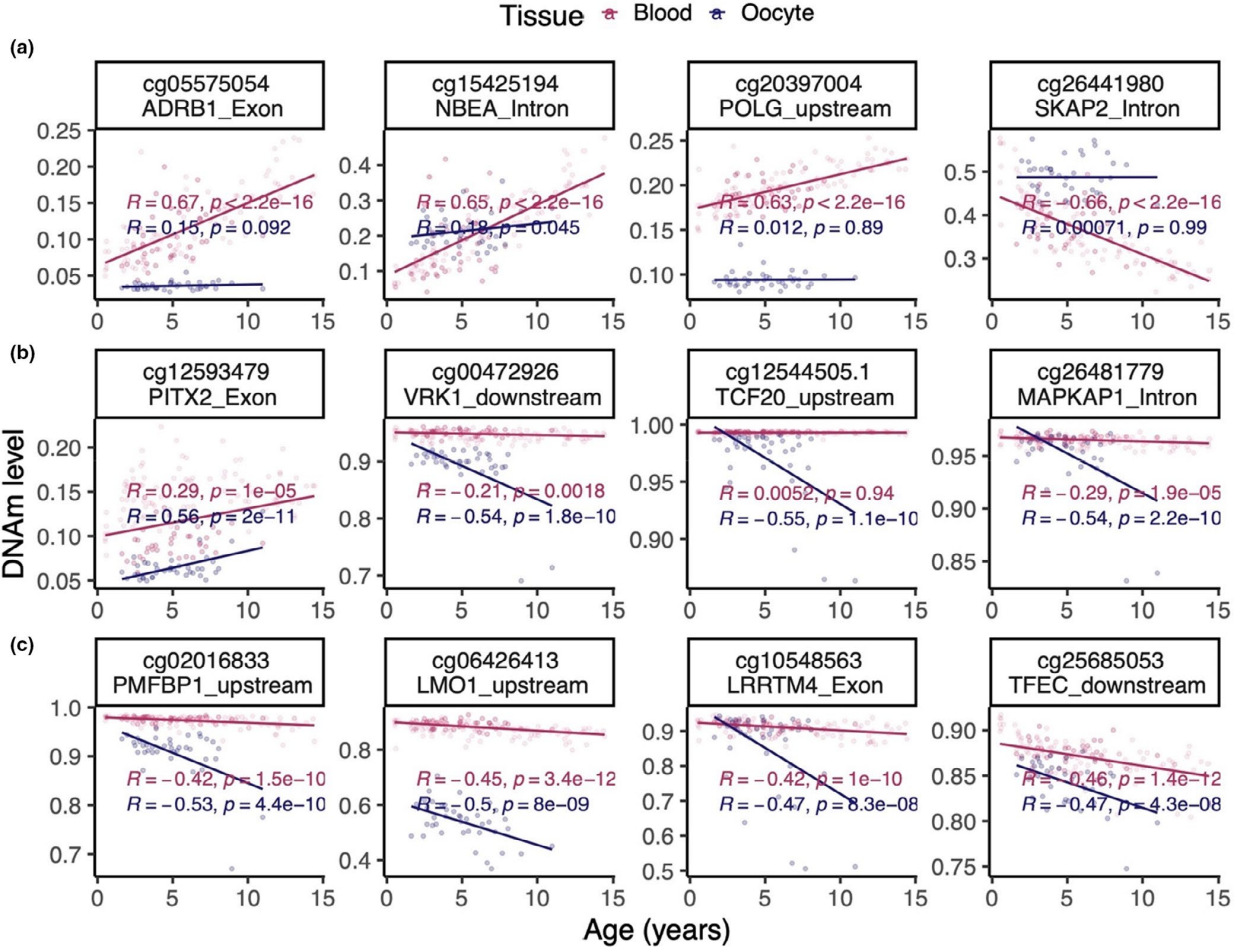
Scatter plots of the most significantly age‐associated CpGs in cattle blood and oocytes. (a) Top four DNAm age changes in aging blood. cg15425194 and cg05575054 were also identified by meta‐analysis. (b) Top DNAm age changes in aging oocytes. (c) Additional DNAm changes identified by meta‐analysis

An epigenome‐wide association study (EWAS) on chronological age in bovine blood and oocyte samples was also conducted. The results revealed that blood and oocytes have distinct age‐dependent DNA methylation changes. Individual CpGs exhibited highly significant age correlations in blood and oocytes (Figure 3a). Results from the EWAS showed that individual CpGs in cattle blood (Figure 3a), oocytes (Figure 3b) were highly associated with age. Oocytes and blood have distinct sets of age‐related CpGs; with oocytes having considerably fewer age‐related CpGs (Figure 3, Figure S6A,C). Although more than 500 CpGs were hypermethylated in blood (at a significance threshold *p* < 10^−4^), only 71 and 70 CpGs were hypermethylated and hypomethylated, respectively, in oocytes at the same significance threshold (Figure 3b‐c). This striking difference in the number of age‐related CpGs between tissue types was also observed when restricting the analysis to blood and oocyte samples from the same animals (Figure S6). The top DNAm changes are as follows: hypermethylation in *NBEA* intron in blood (Figure 4a), and hypomethylation in *TCF20* downstream region in oocytes (Figure 4b). In the meta‐analysis of blood and oocytes, the top DNAm change was also hypermethylation in NBEA intron (Figure 3a, Figure 4c). One of the top DNAm changes in oocytes was near the *DENND1A* gene (Table 1, chromosome 11). Genetic variants in *DENND1A* have been suggested to play a role in susceptibility to polycystic ovary syndrome (*PCOS*), and the most common endocrine disease among premenopausal women. PCOS is a complex disorder characterized by infertility, hirsutism, obesity, various menstrual disturbances, and enlarged ovaries studded with atretic (degenerated) follicles (Eriksen et al., 2013; Goodarzi et al., 2012).

**TABLE 1:** Top 40 negatively and positively age‐associated CpGs in oocyte

CGid	SYMBOL	Region	Chromosome	*Z* score	*p*	Conservation in human
cg12593479	PITX2	Exon	6	5.32	1.1E‐07	different
cg04734735	HOXD8	Exon	2	5.17	2.3E‐07	different
cg20627174	EN1	Intergenic_downstream	2	5.14	2.7E‐07	conserved
cg21811143	EN1	Intergenic_downstream	2	4.98	6.3E‐07	conserved
cg09009312	DENND1A	Intron	11	4.97	6.7E‐07	conserved
cg03461257	DENND1A	Intron	11	4.89	1.0E‐06	conserved
cg21215767	EN1	Intergenic_downstream	2	4.62	3.8E‐06	conserved
cg17892435	PITX2	Intron	6	4.62	3.9E‐06	conserved
cg19688271	MKI67	Intergenic_upstream	26	4.53	6.0E‐06	different
cg14467682	EN1	Intergenic_downstream	2	4.52	6.3E‐06	conserved
cg17245416	NOG	Intergenic_downstream	19	4.49	7.0E‐06	conserved
cg09671306	SYNGAP1	Intron	23	4.48	7.6E‐06	conserved
cg22222486	FOXO6	Intergenic_upstream	3	4.47	8.0E‐06	conserved
cg12911542	HOXA6	Promoter	4	4.45	8.6E‐06	different
cg11375458	HOXD8	Intergenic_downstream	2	4.44	9.1E‐06	different
cg25379954	PROX1	Exon	16	4.42	1.0E‐05	conserved
cg24949381	DMRT1	Intron	8	4.39	1.1E‐05	conserved
cg25528941	MAFF	Exon	5	4.39	1.2E‐05	conserved
cg11040386	IFFO2	Exon	2	4.38	1.2E‐05	conserved
cg25162386	MAFF	Exon	5	4.37	1.2E‐05	conserved
cg17414489	GPC3	Intron	X	−4.47	7.8E‐06	conserved
cg26899365	ARID1B	Intron	9	−4.50	6.9E‐06	conserved
cg08676154	ERI3	Intron	3	−4.50	6.7E‐06	conserved
cg15419333	ZFHX3	Intergenic_downstream	18	−4.50	6.7E‐06	different
cg22825448	DMBX1	Promoter	3	−4.64	3.4E‐06	conserved
cg06426413	LMO1	Intergenic_upstream	15	−4.70	2.6E‐06	different
cg24981538	ARHGAP15	Intron	2	−4.72	2.4E‐06	conserved
cg11393097	MAPKAP1	Intron	11	−4.74	2.1E‐06	conserved
cg06894182	POU4F3	Intergenic_downstream	7	−4.75	2.0E‐06	conserved
cg22968128	ETV5	Intron	1	−4.77	1.9E‐06	conserved
cg21621926	URI1	Intergenic_downstream	18	−4.78	1.8E‐06	conserved
cg22158106	MAPKAP1	Intron	11	−4.78	1.7E‐06	conserved
cg12544505.2	TCF20	Intergenic_upstream	5	−4.79	1.7E‐06	conserved
cg11133252	TCF20	Intergenic_upstream	5	−4.80	1.6E‐06	conserved
cg27135106	URI1	Intergenic_downstream	18	−4.85	1.3E‐06	conserved
cg20814160	FAM193B	Exon	7	−4.93	8.1E‐07	conserved
cg02016833	PMFBP1	Intergenic_upstream	18	−5.15	2.7E‐07	different
cg26481779	MAPKAP1	Intron	11	−5.28	1.3E‐07	conserved
cg00472926	VRK1	Intergenic_downstream	21	−5.28	1.3E‐07	different

**FIGURE 5:**
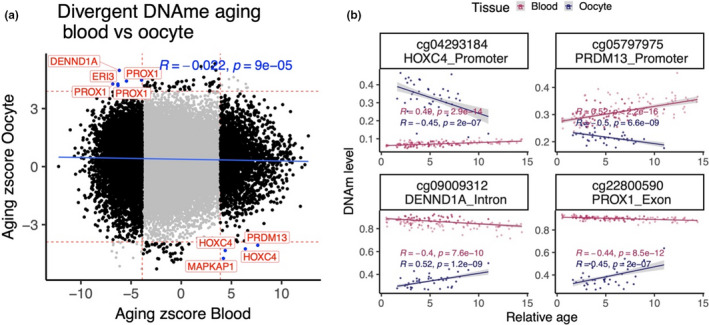
DNAm aging has low correlation between blood and oocyte. (a) Sector plot of DNAm aging in blood and oocyte samples. The red lines indicate *p* < 1e‐4 in each EWAS. The black dots are the CpGs with differential aging pattern between blood and oocyte. The CpGs with significant divergent aging pattern are colored blue and labeled by gene symbol. (b) Scatter plot of top four CpGs with divergent DNAm aging pattern

Across all CpGs, age effects in cattle blood exhibited a weak negative correlation (*r* = −0.02) with those in oocyte (Figure 5a). Although a conservative *p*‐value threshold of 10^−4^ was used to define age‐related CpGs, findings are qualitatively the same for other significance thresholds. Striking differences between blood and oocytes were observed among the significant age‐related CpGs. First, age‐related CpGs in gene promoters exhibited positive age correlations in blood but not in oocytes (odds ratio = 23.3, hypergeometric test *p* = 7.2 x 10^−7^, Figure 3b). Second, none of the age‐related CpGs were shared between blood and oocytes (Figure 3c). For CpGs outside of CpG islands, the mean methylation level in oocytes was substantially lower than that of blood (Figure 3d). The difference was less pronounced for island CpGs (Figure 3d). In blood, island CpGs exhibited substantially higher positive age associations than CpGs outside of islands (Figure 3e). This well‐known pattern (Horvath, 2013) could *not* be observed in oocytes. Further, non‐island CpGs in oocytes appear to be refractive to demethylation with age (Figure 3e), which may reflect that their ground‐state of methylation was already relatively low and could not be further reduced. Few CpGs were even identified with a divergent DNAm aging patterns between blood and oocyte (Figure 5a,b).

Enrichment of human tissue‐specific epigenome states suggested that hypermethylated CpGs in blood are located near bivalent/poised transcription start sites, flanking bivalent TSS, bivalent enhancers, and repressed polycombs (Figure S7). These features were similarly observed in humans (Horvath, 2013). Histone 3 marks for blood hypermethylated CpGs included H3K27me3, H3K4me1, and H3K9me3. Age‐related CpGs found in oocytes were not enriched for any epigenetic signatures of tissue type. By contrast, age‐related CpGs found in blood were, as expected, enriched for epigenetic signatures of blood (Figure S7). Enrichment analysis of genes proximal to the top age‐related CpGs in blood and oocyte is shown in Figure S2.

## 3 DISCUSSION

This study showed that DNAm aging between blood and oocyte has low correlation and even diverges in polar direction in few loci. Interestingly, CpGs within *DENND1A* intron were among those that changed with age in both oocyte and blood, but while they became increasingly hypomethylated with age in blood, they were hypermethylated in aging oocytes (Figure 5b).

Recent evidence suggests that SIRT1 plays a role in oocyte function in older ages (Iljas et al., 2020). The mammalian array used in the current study did not cover the *SIRT1* gene in the cattle genome, but CpGs in the 1 Mb flanking region of the *SIRT1* gene could be investigated (Figure S8). Several CpG probes downstream of *SIRT1* were hypomethylated with age in both blood and oocytes. Interestingly, a probe upstream of *SIRT1* was only hypomethylated with age in oocytes but not in blood.

Methylation of DNA plays a well‐known role in genomic imprinting, which is one of the most important events in the development and maturation of oocytes. The mammalian array covered 23 CpG probes proximate to seven known parental imprinted genes (DCN, GNAS, PEG10, NAA60, MES, PLAGL1, and SFMBT2) in cattle. None of the observed DNAm aging loci in blood or oocytes overlapped with these seven imprinted genes.

The strong difference in multiple characteristics of age‐related CpGs between blood and oocytes may be explained by the fact that blood cells are continually renewed while oocytes are formed during embryogenesis. Antral follicles differ substantially from primordial follicles, as is evident from the wave‐like pattern of DNA methylation. Hence, we purposefully choose small antral follicles in the germinal vesicle stage which were already in the cohort of follicles selected for further development. By this we ensured that the follicles reflected a particular and defined developmental competence (potential to reach the stage of a Graffian follicle). Further research is needed to investigate whether our presented oocyte clocks lend themselves for direct application to human oocytes.

Given the unique biology of oocytes, it is remarkable that one can build highly accurate oocyte‐based epigenetic clocks including dual‐tissue clocks that apply to both oocytes and blood. If the oocyte epigenetic clocks turn out to be indicators of the fitness of oocytes, it would suggest the existence of a maternal age‐dependent program that specifically alters the DNA methylation state of the oocyte. The endocrine system, which changes with age, could relate to this program. Indeed, age‐related hormonal changes are very well‐documented, and it is worth noting the recent report on the rejuvenation effects of human growth hormone (Fahy et al., 2019). Regardless of the actual mechanism, which requires empirical elucidation, the potential importance of age‐related DNA methylation changes on oocyte DNA cannot be ignored. The epigenetic clocks presented herein for oocytes are expected to be useful for finding answers to many questions that range from fecundity to evolutionary selection.

## 4 CONCLUSION

The primary objective of the study was to develop and apply epigenetic biomarkers of aging for oocytes from cattle as a model for human oocytes. Although a dual‐tissue epigenetic clock was developed that was predictive of age in both blood and oocytes, the fundamental aging properties of these two sample types were found to differ substantially. The rate of epigenetic aging was found to be slower in oocytes compared to blood; however, oocytes appeared to begin at an older epigenetic age. The differences in epigenetic aging effects between oocytes and blood were observed at the level of individual CpGs and cumulatively at the level of single‐tissue epigenetic clocks. The epigenetic clocks for oocytes are expected to address questions in the field of reproductive aging, including the central question: how to slow aging of oocytes.

## 5 EXPERIMENTAL PROCEDURES

### 5.1 Ethical authorization and animals

All animal procedures were carried out in accordance with the relevant guidelines at each institution. Specifically, procedures related to sample collection in Poland followed the EU Directive of the European Parliament and the Council on the protection of animals used for scientific purposes (22 September 2010; No 2010/63/EU), Polish Parliament Act on Animal Protection (21 August 1997, Dz.U. 1997 nr 111 poz. 724) with further novelization – Polish Parliament Act on the protection of animals used for scientific or educational purposes (15 January 2015, Dz.U. 2015 poz. 266). Blood and oocyte collection were approved by the Local Ethics Committee for Experiments on Animals, University of Warmia and Mazury in Olsztyn, Poland (Agreement No. LKE.065.27.2019). For animal procedures in the USA, approval from the University of Nebraska Institutional Animal Care and Use Committee was obtained (approval number is 1560). Samples were obtained from 357 female cattle (Bos Taurus). The animals were housed on the Dairy Farm of the Institute of Animal Reproduction and Food Research of Polish Academy of Sciences, (Wielki Las, Poland) and at the Eastern Nebraska Research and Extension Center at the University of Nebraska‐Lincoln (Nebraska, USA). In the present study, samples were collected from Polish Red cattle and from the herd in Eastern Nebraska. In this herd, black Angus or composites of varying percentages of Simmental x Angus (black) or Red Angus where used as blood donors. Animals were free of Bovine Herpesvirus Type 1, Bovine Viral Diarrhea/Mucosal Disease, tuberculosis, and Enzootic bovine leucosis.

### 5.2 Blood collection and further processing

The blood samples from both herds were collected during routine animal management activities, during the routine blood collection for disease prevention. Blood samples were taken only from cows in the luteal phase of the estrous cycle. Blood was collected into 8 ml PAXgene Blood DNA Tubes (Quiagen, Cat No. 761115) and stored at −80℃ until the shipment (USA Veterinary Permission Nr 138809) to the UCLA Technology Center for Genomics & Bioinformatics (Los Angeles, USA) for further analyses.

### 5.3 Oocyte Collection and further processing

In total, 80 Bovine ovaries were collected immediately *postmortem* from 40 cows which were selected for routine culling due to management reasons. All cows were in the luteal phase of the estrous cycle. Before the isolation of both ovaries from each cow, blood samples were also collected into 8 ml PAXgene Blood DNA Tubes (Quiagen, Cat No. 761115) to generate both sample types from one donor. Isolated ovaries were kept on ice and immediately transported to the laboratory. Afterward, immature bovine cumulus–oocyte complexes (COCs) were recovered by aspirating ovarian follicles in the diameter of 2–8 mm. Cumulus cells were removed from COCs by pipetting them for 5 min in a Petri dish containing 500 μl of Phosphate Buffered Saline (PBS) with 0.1% hyaluronidase (Sigma‐Aldrich). Denuded oocytes from every donor were pooled (10–15 immature oocytes/ovary) and then processed for genomic DNA isolation.

### 5.4 Genomic DNA isolation

Genomic DNA was isolated from blood and oocyte samples using the DNeasy Blood and Tissue Kit (Qiagen, Cat No. 69506) and subsequently bisulfite converted using the EZ DNAMethylation Kit (ZymoResearch). Bisulfite‐treated samples were processed using the custom array.

### 5.5 DNA methylation profiling

All methylation data were generated using a custom Illumina methylation array (HorvathMammalMethylChip40) based on 37492 CpG sites. Out of these 37,492 sites, 1951 were selected based on their utility for human biomarker studies; these CpGs, which were previously implemented in human Illumina Infinium arrays (EPIC, 450 K, 27 K), were selected due to their relevance for estimating human age, human blood cell counts or the proportion of neurons in human brain tissue. The remaining 35,541 probes were chosen due to their location in stretches of DNA that are highly conserved across mammalian species (Arneson et al., 2021).

The particular subset of species for each probe is provided in the chip manifest file at the NCBI Gene Expression Omnibus (GEO) platform (GPL28271). The SeSaMe normalization method was used to define beta values for each probe.

### 5.6 Probe mapping and annotation

The probe sequences were aligned to cattle (*Bos taurus*) ARS‐UCD1.2 genome using QUASR package (Gaidatzis et al., 2015) with the assumption for bisulfite conversion treatment of the genomic DNA. The QUASR (a wrapper for Bowtie2) was run with parameters ‐k 2 ‐‐strata ‐‐best ‐v 3 and bisulfite = "undir” to align the probe sequences to each prepared genome. Following the alignment, the CpGs were annotated based on the distance to the closest transcriptional start site using the Chipseeker package (Yu et al., 2015). A gff file with these was created using these positions, sorted by scaffold and position, and compared to the location of each probe in BAM format. Genomic location of each CpG was categorized as intergenic, 3′ UTR, 5′ UTR, promoter region (minus 10 kb to plus 1000 bp from the nearest TSS), exon, or intron.

### 5.7 Epigenome‐wide association studies of age

The DNAm changes were examined for association with age using the R function "standardScreeningNumericTrait" from the "WGCNA" R package (Langfelder & Horvath, 2008) in each tissue. The results were combined using Stouffer's meta‐analysis method.

Downstream enrichment analysis of the significant differentially methylated positions (DMP) was performed for transcriptional factor (TF) motifs and merged datasets of gene ontology, canonical pathways, upstream regulators, diseases, and phenotypes using the neighboring gene sets. The gene‐level enrichment was done using GREAT analysis (McLean et al., 2010) and human Hg19 background, but pre‐filtered to 22,217 probes that were mapped to the same gene in both human and cattle genomes.

## CONFLICT OF INTEREST

SH is a founder of the non‐profit Epigenetic Clock Development Foundation which plans to license several patents from his employer UC Regents. These patents list SH as inventor. The other authors declare no conflicts of interest.

## AUTHOR CONTRIBUTIONS

P.K. and M.L.S collected the DNA samples and carried out DNA extraction. P.K., S.H, K.R., M.L.S, and A.H drafted the article. J.Z., A.H, C.Z.L, and S.H. conducted the statistical analysis. S.H. and P.K conceived the study.

## CODE AVAILABILITY

Details on the epigenetic clocks can be found in Supplementary Methods.

## Supporting information

Fig S1‐S8

## Data Availability

The data will be made publicly available as part of the data release from the Mammalian Methylation Consortium. Genome annotations of these CpGs can be found on Github https://github.com/shorvath/MammalianMethylationConsortium.
