# Supplementary material for: Epigenetic clock and methylation study of oocytes from a bovine model of reproductive aging
Source: Aging Cell. 2021 Apr 2;20(5):e13349. doi: 10.1111/acel.13349 (PMC8135012; doi:10.1111/acel.13349)
Supplement: Supplementary file 1 — Fig S1‐S8 [file ACEL-20-e13349-s001.docx]

**Supplementary Figure 1.**

**
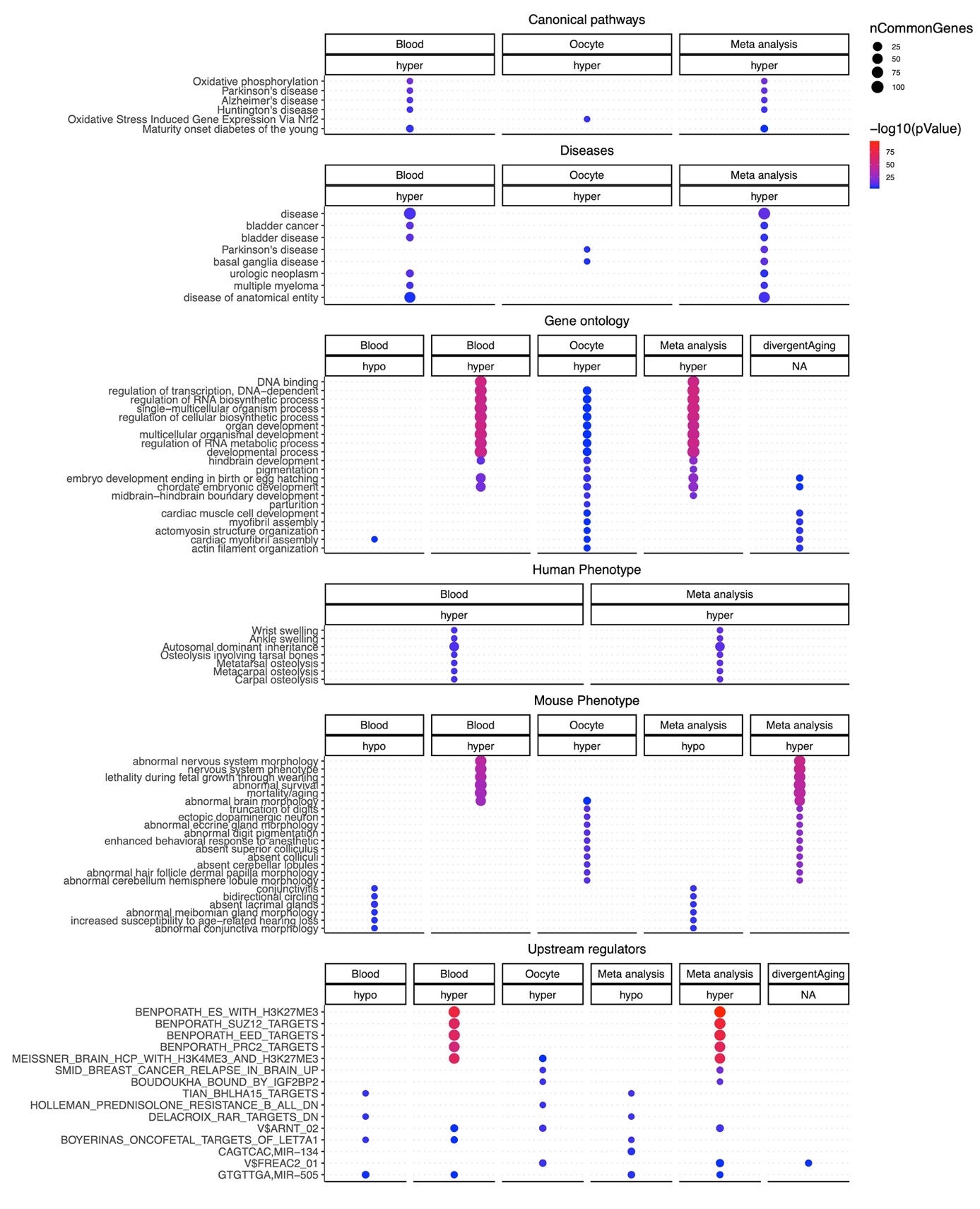
**

**Supplementary Figure 2.**

**Supplementary Figure 3.**


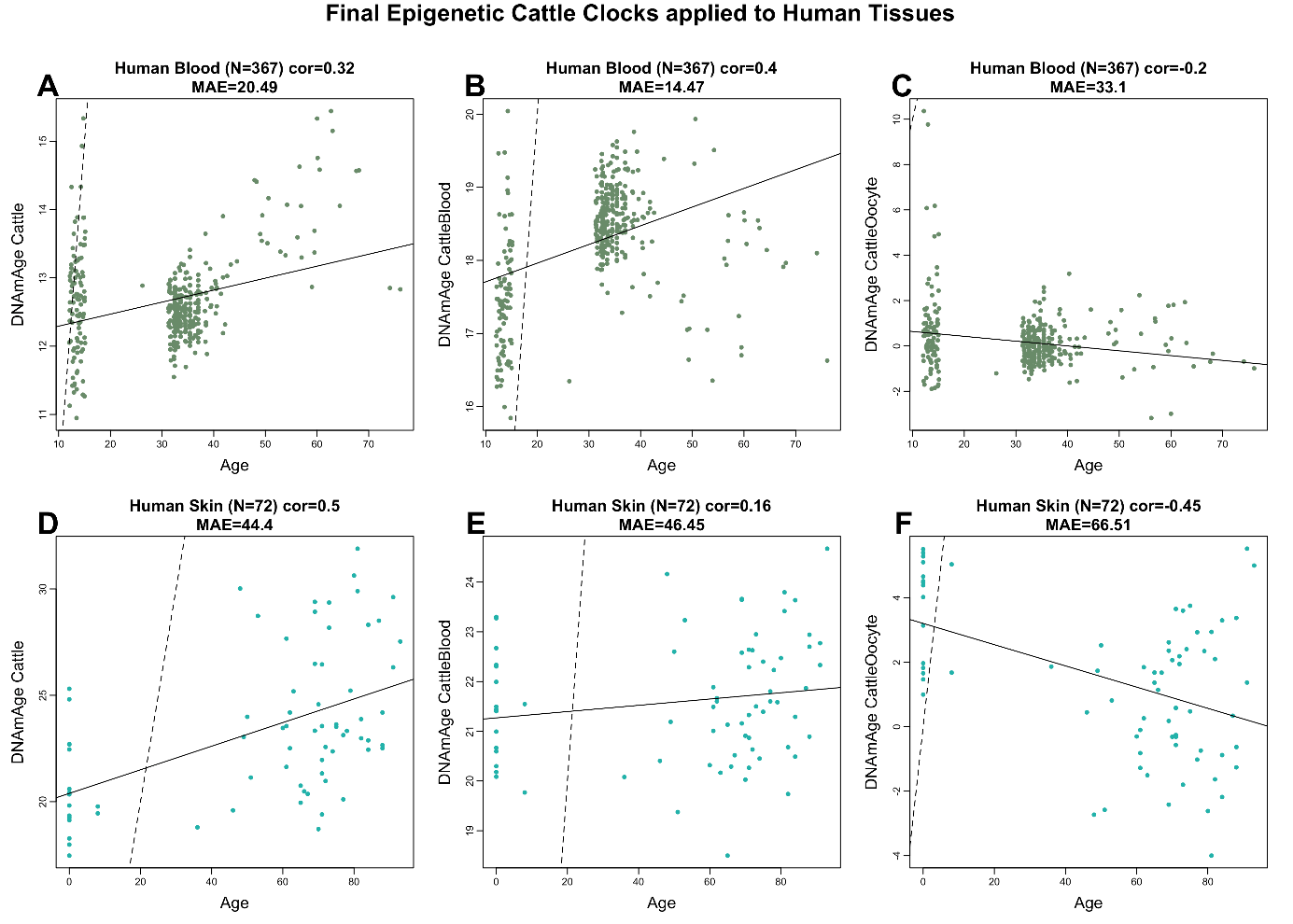
**Supplementary Figure 4.**

**
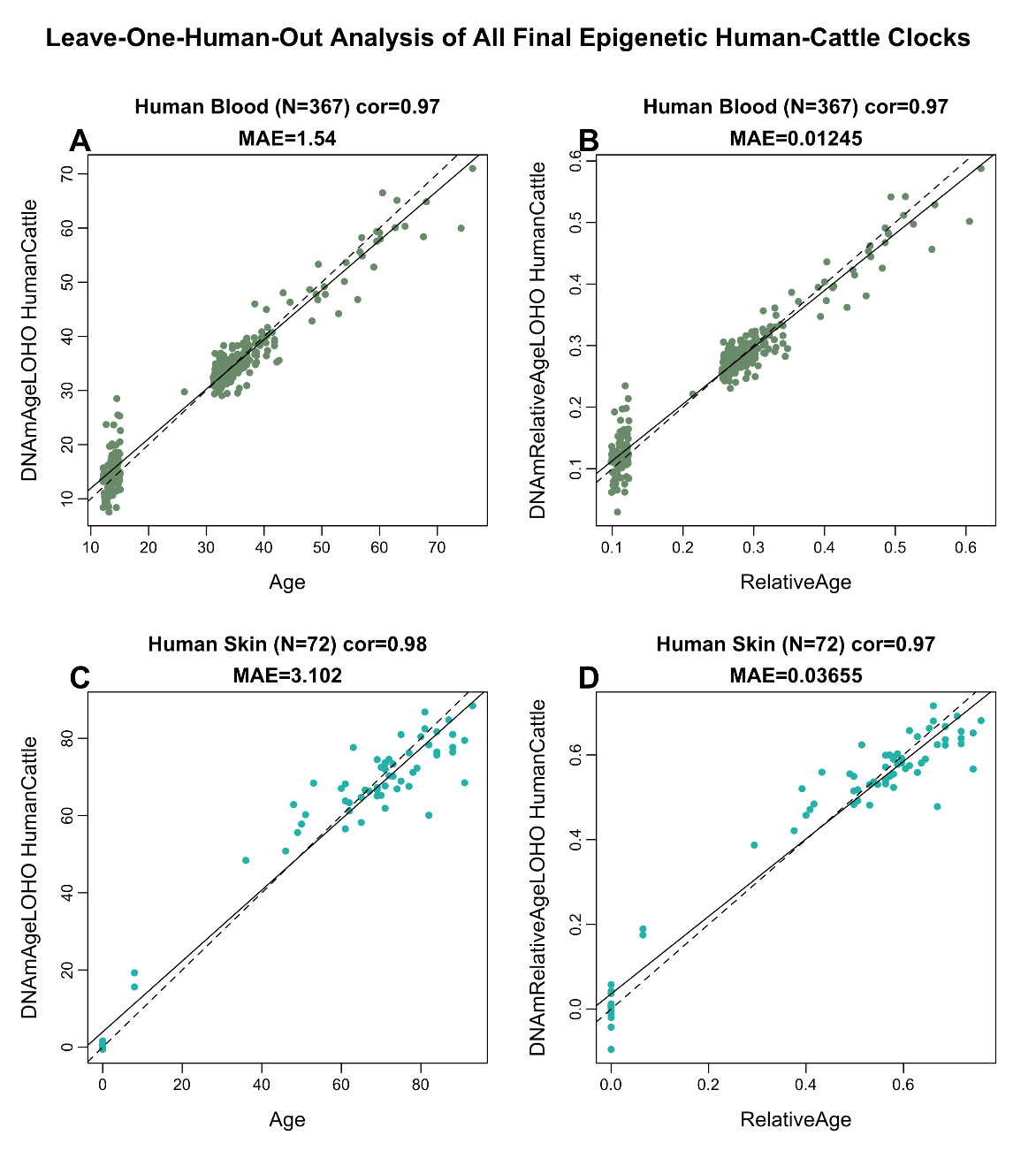
**

**Supplementary Figure 5.**


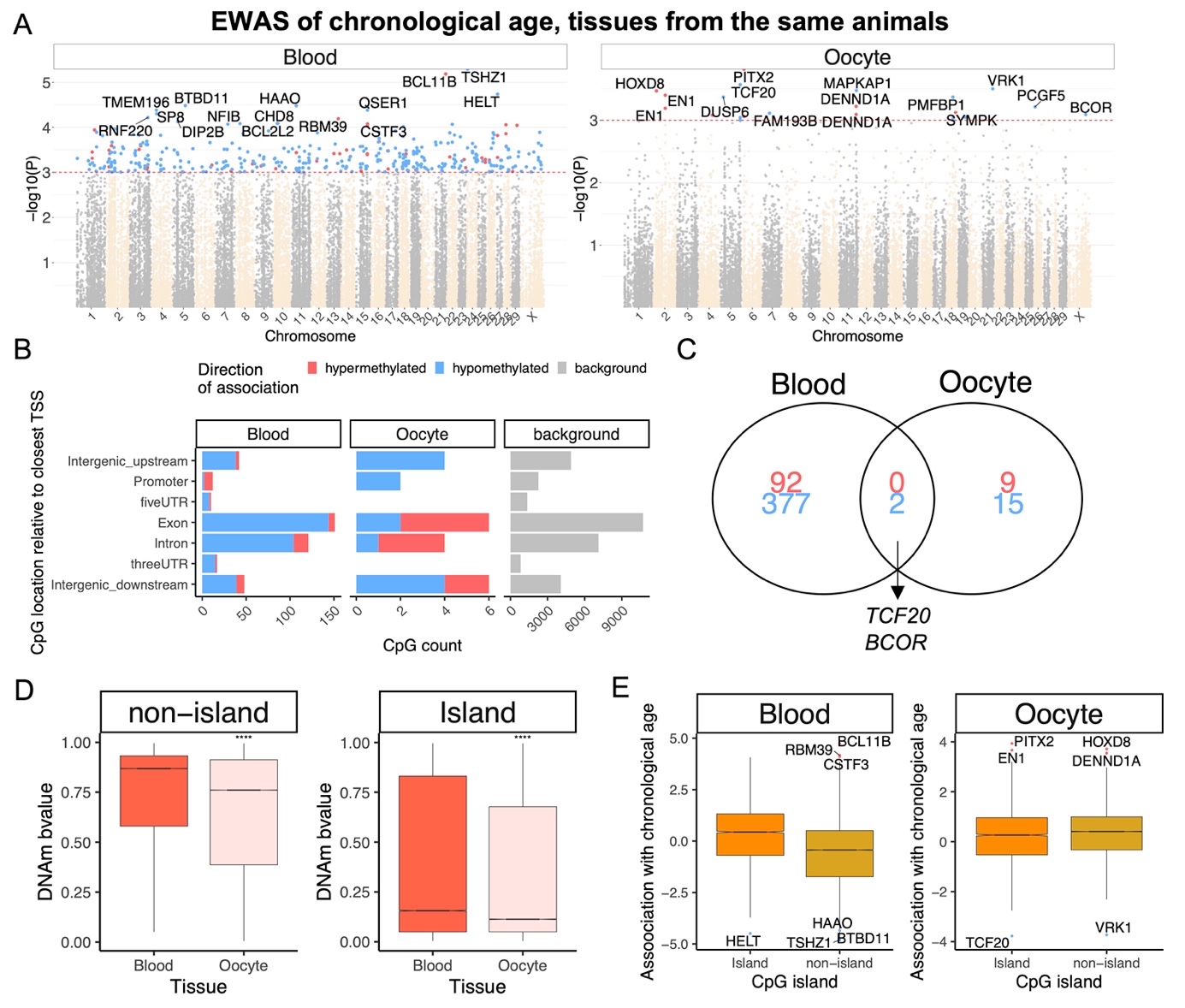


**Supplementary Figure 6.**

**
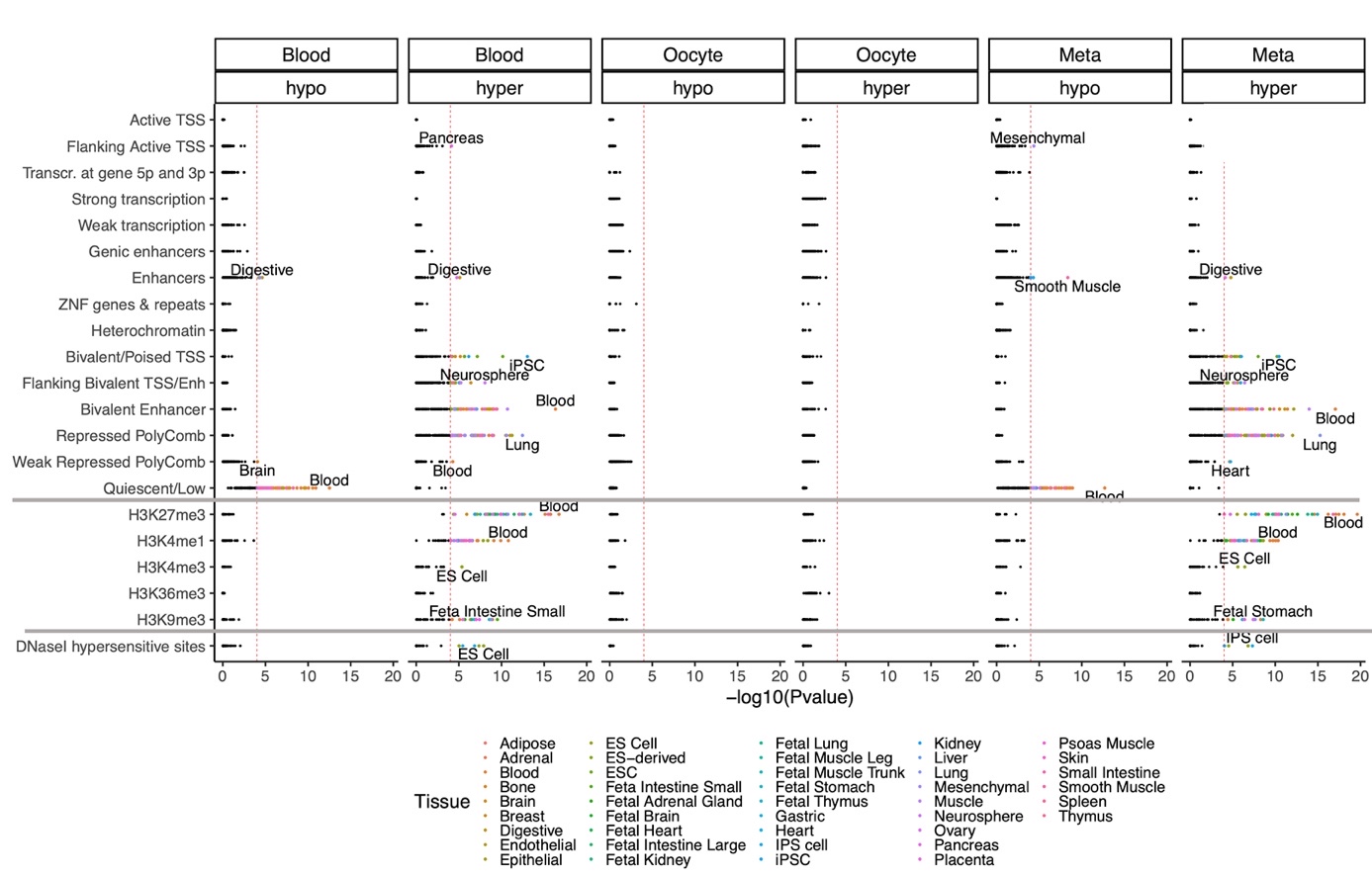
**

**Supplementary Figure 7.**

**
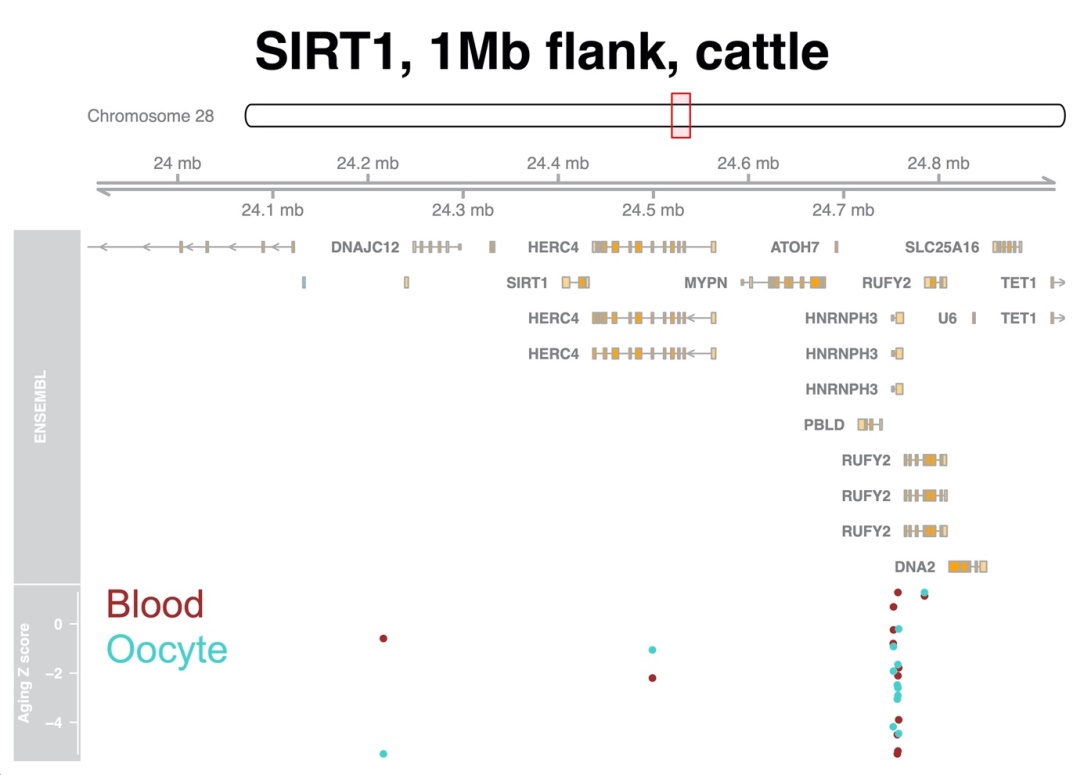
**

**Supplementary Figure 8.**
